# Supplementary material for: Chemical Chaperone 4-PBA Mitigates Tumor Necrosis Factor Alpha-Induced Endoplasmic Reticulum Stress in Human Airway Smooth Muscle
Source: Int J Mol Sci. 2023 Oct 31;24(21):15816. doi: 10.3390/ijms242115816 (PMC10649207; doi:10.3390/ijms242115816)
Supplement: Supplementary file 1 [file ijms-24-15816-s001.zip › ijms-2670472-supplementary.pdf]

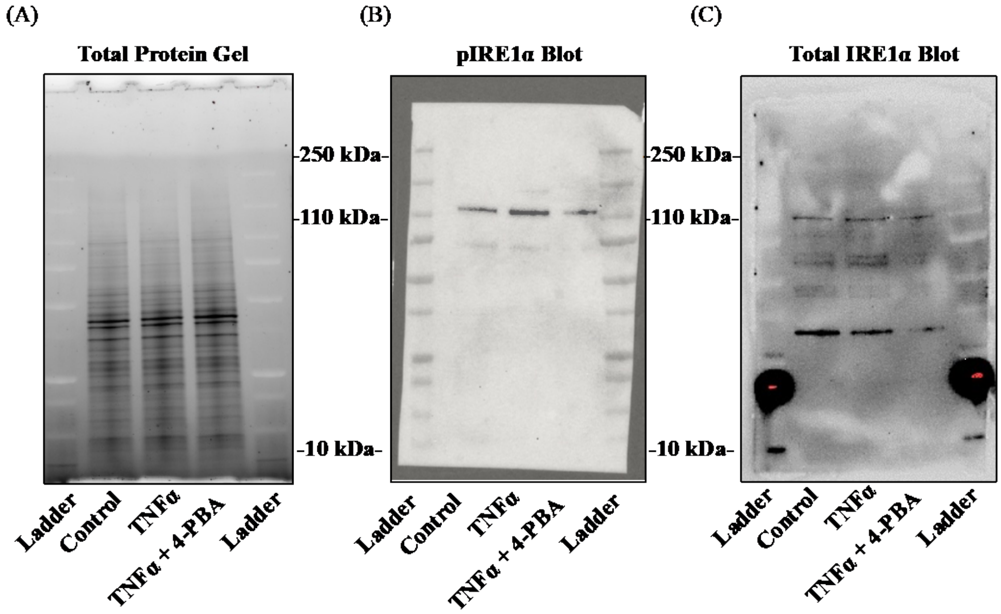

Supplemental Figure S1. Complete Total protein gel and blots used for Western Blot analysis shown in Figure 4. Total protein was quantified using a criterion TGX stain free precast gel (A). Trans-Blot Turbo transfer pack (BioRad catalog #1704157) was used to transfer proteins from the gel to a Midi format PVDF single application membrane which was blocked and incubated with (B) pIRE1αS724 antibody (ab124945) or (C) total IRE1α antibody (NB100-2324). Precision Plus All Blue Protein Standard was used as the ladder (BioRad catalog #161-0373). All imaging was done on a ChemiDoc MP imaging system.

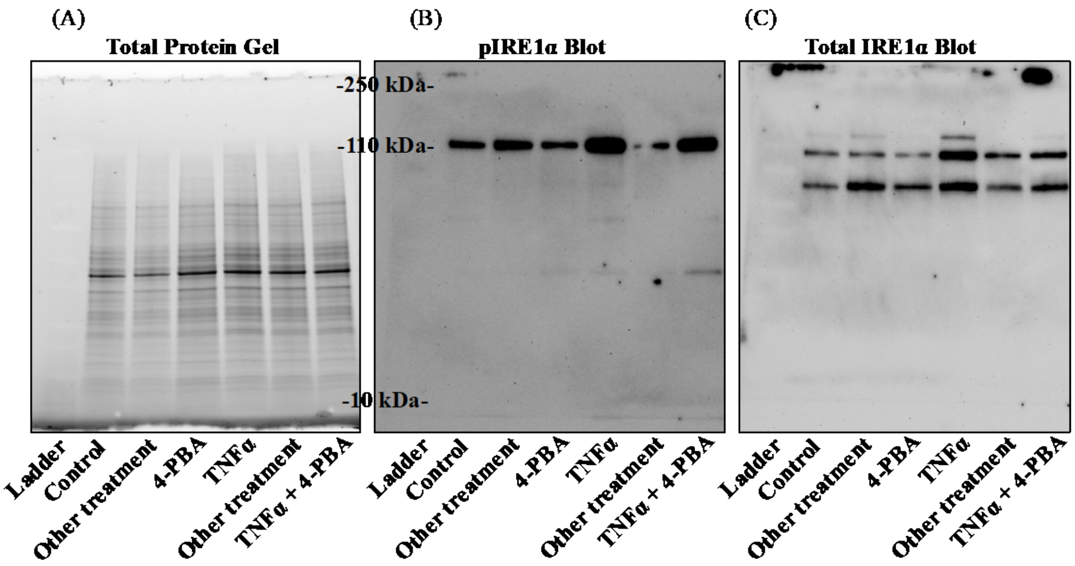

Supplemental Figure S2. Complete gel and blots used for Western Blot analysis to determine the effect of 4-PBA treatment alone. Total protein was quantified using a criterion TGX stain free precast gel (A). Trans-Blot Turbo transfer pack (BioRad catalog #1704157) was used to transfer proteins from the gel to a Midi format PVDF single application membrane which was blocked and incubated with (B) pIRE1αS724 antibody (ab124945) or (C) total IRE1α antibody (NB100-2324). Precision Plus All Blue Protein Standard was used as the ladder (BioRad catalog #161-0373). All imaging was done on a ChemiDoc MP imaging system.

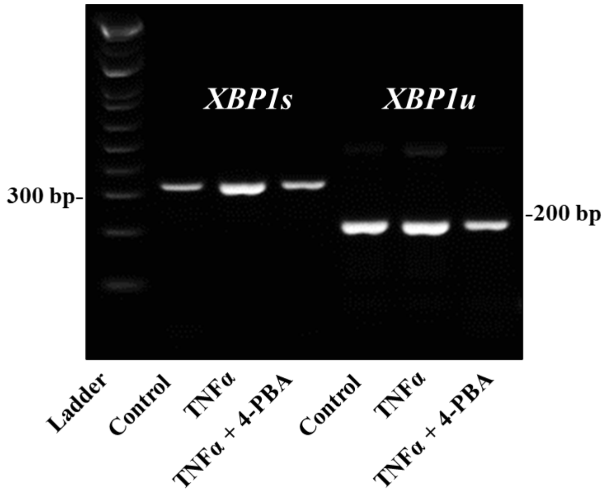

Supplemental Figure S3. Complete gel used for mRNA PCR analysis and used for Figure 5. mRNA bands were quantified using a ChemiDoc MP imaging system. An Invitrogen 1 Kb Plus DNA Ladder (REF10787018) was used as a marker.

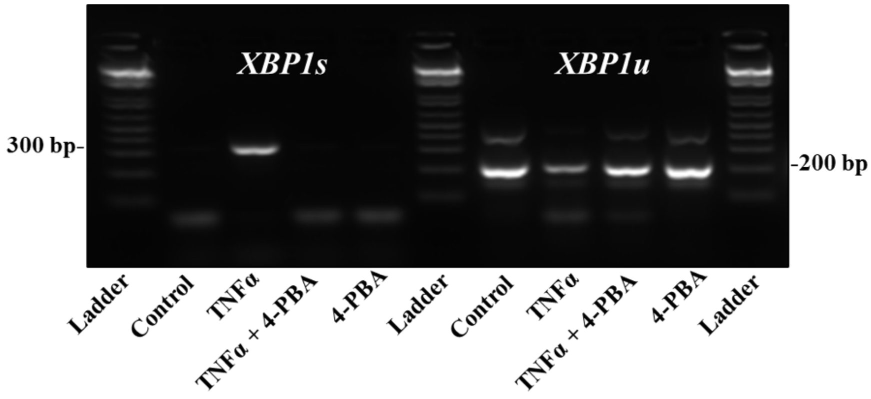

Supplemental Figure S4. Complete gel used for mRNA PCR analysis to examine the effect of TNFα, TNFα + 4-PBA and 4-PBA on XBP1 splicing. mRNA bands were quantified using a ChemiDoc MP imaging system. An Invitrogen 1 Kb Plus DNA Ladder (REF10787018) was used as a marker.
